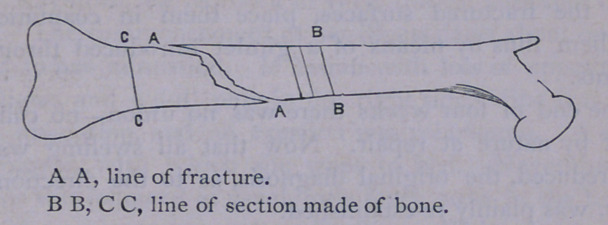# A Case of Non-Union of Fracture of the Femur, Caused by the Interposition of Muscle between the Fragments

**Published:** 1871-06

**Authors:** C. T. Parkes

**Affiliations:** Chicago


Article IV.—*A Case of Non-Union of Fracture of the Femur, caused by the interposition of Muscle between the fragments.* By C. T. Parkes, M.D., Chicago.

The many points of interest in connection with the case about to be recorded has led me to think it worthy of publication. I was called to attend J. M——, March 16th, 1870, who had been injured by being buried beneath a mass of frozen coal. I requested Prof. M. Gunn to accompany me. We reached the patient's residence four hours after the accident had happened, and upon examination found an oblique fracture of the right femur, a little below the junction of the lower with the middle third of the bone. We immediately applied to the limb proper dressings, making extension with adhesive straps, weight and pulley, with a long external splint to prevent rotation, and short splints to the thigh. An accurate examination as to the exact seat and condition of the fracture was not attempted, but deferred until the subsidence of swelling, etc., should allow a thorough and complete diagnosis.

On the third day after the injury, I removed all the dressing, found the effusion very much reduced, and made a careful examination of the injured limb.

I found very great freedom of motion at the seat of fracture, but failed to obtain one particle of crepitation, either during extreme extension, perfect relaxation or rotation, and finally came to the conclusion—

1st. That there was an oblique fracture of the bone at the point mentioned. The line of obliquity differed from that of any fracture which my experience could recall. A pointed, well-marked spicula seemed to be separated from the outside of the shaft of the bone, giving at first the idea of a very oblique fracture; but upon tracing the spicula upward for one-half or three-quarters of an inch, it abruptly terminated in a transverse ledge of bone extending across the shaft for the greater part of its diameter, and then shelving off into a depression on the inner and under aspect of the bone. The following rough cut will illustrate the description as given.

A A, line of fracture.

B B, C C, line of section made of bone.

Subsequent events enabled me to demonstrate positively that such was the direction of the fracture.

2d. That some tissue or other had been thrown between the fractured ends, by the force of the injury, and this also was proven by actual demonstration.

A section of this femur placed in my hands showed me the upper end of the lower fragment, completely enveloped by a mass of muscle, half an inch thick and an inch broad.

After the examination was made, I redressed the limb with strips of adhesive plaster, extending up to the seat of fracture, weight and pulley as before. Prof. Gunn saw the case with me again on the next day, and confirmed the diagnosis which I had made. All efforts to bring the broken ends into coaptation were fruitless.

As there was no absolute certainty about the reason for the absence of crepitation; as the want of it might be due to effusion merely; as the normal length, etc., of the limb could be easily maintained, and as it seemed unwarrantable to convert a *simple* fracture of the thigh into a *compound one*, on such uncertain conditions, especially where the soft parts were so terribly contused as they were in this case, it was deemed advisable to await the efforts of nature at repair, for a time at least. So the patient was made acquainted with the condition of the injury, and told that in all probability the fracture would not unite without an operation, but, for the reasons already given, we thought it better to treat the case as an ordinary one for at least four or five weeks, in order to give nature every opportunity to prove our suppositions correct, or to remove the obstructions to coaptation by absorption. The ends of the fractured bone were bound tightly together by means of compresses and splints to the thigh. The operation deemed necessary, and what we contemplated doing, was to cut down upon the fracture from the outside of the thigh, turn the ends out, remove entire whatever tissue might be found between them, freshen the fractured surfaces, place them in coaptation, and retain them thus by means of a gimlet introduced through the fragments.

At the end of four weeks there was no union—no callus—no attempt by nature at repair. Now that all swelling was completely reduced, the original diagnosis, as to the direction of the fracture, was plainly re-established.

The patient was advised to submit to the operation as described above. He objected, and desired to wait two weeks longer, as he had assured himself that he "felt them knitting together." At the end of the six weeks, another physician of the city was called in consultation, at the request of the patient. I met him at the patient's bedside, and he finally concluded to take the case off my hands, under the supposition, as I understood it, that he could obtain union without an operation of the nature contemplated. Subsequently the doctor kept the patient under treatment for about three weeks, and finally concluded that an operation would be required ere recovery took place. The operation as performed, was just the one to prove that the original diagnosis and suppositions entertained by myself, and confirmed by Prof. Gunn, were in all respects correct. A section, fully an inch and a half long, was sawn from the upper end of the lower fragment, and several sections, amounting in the aggregate to about two inches, were made from the upper fragment. The lower fragment is completely enveloped by a thick layer of muscle, except a small spicula of bone at the extreme upper end.

The patient, as I am told, unfortunately died a few days after the operation, from repeated and severe *secondary hemorrhage.* The bone surfaces present no evidences of absorption, neither have they any deposit of plastic material on them, such as we are told to expect in non-united fractures.

## Figures and Tables

**Figure f1:**